# Influence of clinical expertise and practical experience on transfer accuracy in guided dental implant placement - an in vitro study

**DOI:** 10.1007/s10006-024-01269-4

**Published:** 2024-06-25

**Authors:** Florian Sebastian Reiff, Charlotte Bischoff, Henriette Woelfler, Stefan Roehling

**Affiliations:** 1 Center of Oral Health, University of Greifswald, Greifswald, Germany; 2 Straumann Group, Freiburg im Breisgau, Germany; 3 Straumann Group, Basel, Switzerland; 4 University of Bamberg, Bamberg, Germany; 5 PD Dr. med. dent. / Private Dental Clinic PD Dr. Gahlert and PD Dr. Roehling, Munich, Germany; 6 Clinic for Oral and Cranio-Maxillofacial Surgery, Hightech Research Center, University Hospital Basel, Basel, Switzerland

**Keywords:** Accuracy, Experience, Guided surgery, Dental implant, Static navigation

## Abstract

**Purpose:**

To investigate whether inexperienced users applying a static navigation system can perform in-vitro a fully guided implant placement protocol and achieve similar results in terms of accuracy compared to experienced clinicians.

**Methods:**

Based on 36 identical resin models, a computer-assisted implant planning was performed and a surgical guide was produced accordingly. Three study groups were composed with 12 operators, each: control group with experienced surgeons (DOC), test group 1 with dental technicians (TEC) and test group 2 with non-specialists (OFC). Using a fully guided drilling protocol, two implants were placed into each of the 36 models. Subsequently, the differences between the virtually planned and final implant positions were determined and the transfer accuracy was evaluated.

**Results:**

For the control group DOC, the mean value of axial deviation was 1.90 ± 1.15 degrees, for 3-dimensional deviation at the implant base 0.52 ± 0.33 mm, for 3-dimensional deviation at the implant tip 0.76 ± 0.39 mm and for vertical deviation at the implant tip − 0.11 ± 0.51 mm. For corresponding parameters, the mean values of test group TEC were 1.99 ± 0.87 degrees, 0.42 ± 0.21 mm, 0.68 ± 0.30 mm and − 0.03 ± 0.33 mm and for test group OFC 2.29 ± 1.17 degrees, 0.63 ± 0.35 mm, 0.89 ± 0.43 mm and − 0.24 ± 0.57 mm, respectively. The results did not reveal any statistically significant differences between the control and the 2 test groups (p˃0.05).

**Conclusion:**

The results of the present in-vitro study demonstrated that inexperienced users applying a static navigation system can perform a fully guided implant placement protocol and achieve similar results in terms of accuracy compared to experienced clinicians in this specific in vitro setup.

## Background

The use of static navigation systems in implant dentistry to transfer a virtually planned implant position to the clinical site is established and has been studied very well [1–3]. Predictable treatment results and the reduction of intraoperative complications even with complex cases are considered advantages of this concept [4, 5]. The so-called transfer accuracy (TA) is a decisive set of parameters to evaluate the reliability of guided implant placement systems, determining the difference between the virtually planned and the clinically placed implant position. The TA demonstrates how accurately the virtually planned implant position was transferred to the clinical surgical site. The availability of static navigation systems poses the question if users with little or even no experience in implant dentistry can achieve good clinical results, i.e., high accuracy with the help of drilling guides. Only few scientific studies investigated this relation and their results are controversial. For example, clinical and in vitro studies reported that less experienced users benefit from the use of drill templates and significantly improve accuracy values [6, 7]. In contrast, clinical studies found that the degree of experience is not a significant factor influencing the accuracy [8–10]. It was also reported that inexperienced users achieved inferior results in terms of TA [11].

This shows that no clear conclusion can be drawn and further investigation is needed. Thus, the aim of this in-vitro study was to assess the influence of the operator´s clinical expertise and practical experience on TA in guided dental implantation.

## Methods

The experiments were conducted using maxillary polyurethane models of the type Maxilla S1, (GOS® GmbH, Northeim, Germany). After a quality control, a reference model was determined and used for the creation of a 3-dimensional X-ray image using cone beam computer tomography (CBCT). The parameters of the CBCT scanner Veraview X800 (J. MORITA® MFG. CORP., Kyoto, Japan) were set as follows: Voltage 79.0 kV; Amps 1.9 mA; exposure time 9.4 s; original angle 0.0°; FOV 88.125 × 88.125 × 80.500 mm; voxel size 0.125 mm, slice thickness 1.000 mm, slice interval 1.000 mm. Subsequently, the obtained data set was exported as Digital Imaging and Communications in Medicine (DICOM) file format with maximum resolution. The reference model was then scanned with a desktop scanner (Straumann® Desktop Scanner 7-Series, Dental Wings® GmbH, Chemnitz, Germany) and the resulting surface scan was exported as a Standard Tessellation Language (STL) file. Both data sets were superimposed using a planning software (coDiagnostiX®, Dental Wings® GmbH, Chemnitz, Germany) and virtual implant planning was performed. One implant was planned as a single-tooth gap in region 15 and another implant as a free-end situation in region 26. Following that, the drill template was designed and manufactured by an experienced user using the Objet 260 3D printer (Stratasys® Ltd., Rehovot, Israel). The material OBJET MED610 Biocompatible, Clear, (Stratasys® GmbH, Rheinmünster, Germany) validated for this process was used. Original Straumann® T-sleeves, Ø 5 mm, height 5 mm made of steel (Straumann Group, Basel, CH) were used as drill sleeves.

Thirty-six test persons were included in the study, forming three equal groups of operators (Table 1).

**Table 1:**
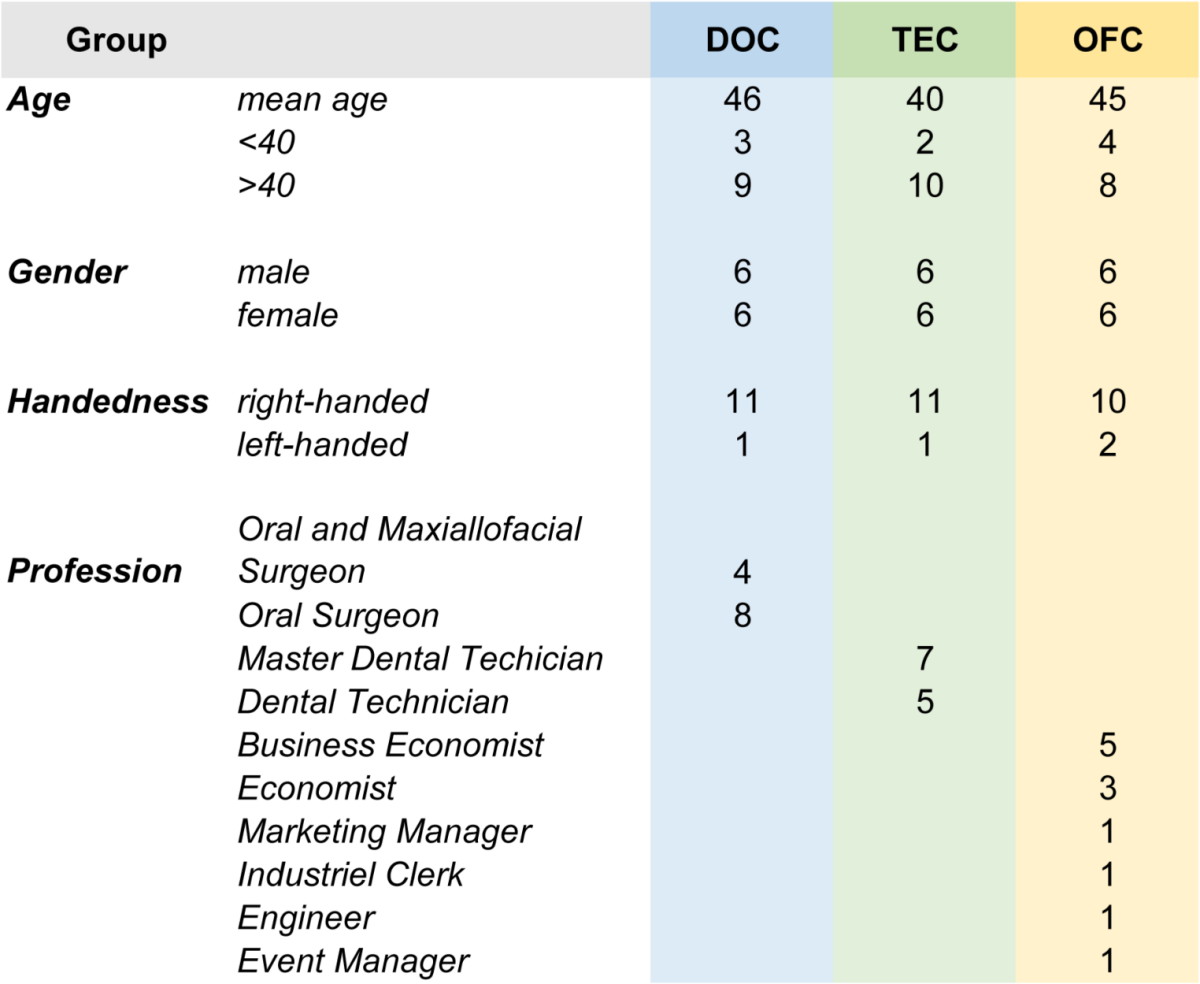
Demographic data of participating subjects

All groups consisted of 50% female and 50% male operators. The control group (DOC) consisted of eight oral surgeons and four oral and maxillofacial surgeons. Both professional titles represent a high level of specialized expertise and practical clinical experience in the field of implant dentistry and surgery, respectively. Test group 1 (TEC) consisted of five dental technicians and seven master dental technicians. Dental technicians have limited theoretical knowledge but no clinical or practical experience with static navigation systems. The test group 2 (OFC) consisted of twelve office workers. The operators of this group were non-clinicians and have neither dental nor dental technical training. Conducting the experimental implant placement was the first contact with implant dentistry resp. guided surgery for them.

### Implementation of the experimental implant placement

All operators performed the experimental implant placement separately under the same conditions and were individually instructed by the investigator. After a technical briefing on the use of the surgical motor and the contra-angle handpiece, the experiment was carried out. A training video (Straumann® Guided Surgery – Leading Your Way In Your Treatment, Straumann Group, Basel, CH) provided by the manufacturer of the implant system showing the clinical application of the guided-implant systems was shown to the operators. In this way, the individual steps of the experimental implant placement were successively demonstrated. Afterwards, the operators autonomously practically performed the demonstrated steps and were supervised by the investigator. The investigator documented the drilling procedures and implant placements of each operator. However, he did not influence this procedure by any kinds of comments or practical interactions.

A total of 72 dummy implants with a tapered design, with a diameter of 4.1 mm and a length of 10 mm (Straumann® BLT implants, Roxolid® SLActive, Straumann Group, Basel, CH) were used for the experimental implant placement. Each operator placed the implants into a separate jaw model, but all operators used the same drill template. A surgical motor with contra-angle handpiece was used for preparation, profile drilling and tapping. Implants were inserted manually using the surgical hand ratchet from the surgical cassette provided. The drilling protocol was performed according to the specifications of the manufacturer’s instructions. The surgical instruments used were the Straumann® Guided Surgery Basic Cassette (Straumann Group, Basel, CH) for guided implant placement. The drills and the profile drill of this system have a physical depth stop. The drills were replaced after 10 times of use according to the manufacturer’s recommendation. For the experimental implant placement, the operators used the Straumann® Surgical Motor Pro with the contra-angle handpiece Ti-Max X-SG20L (NSK® NAKANISHI INC., Tochigi, Japan). For implant placement, the surgical motor settings for the drills with diameters of 2.2 mm, 2.8 mm, and 3.5 mm were set to 800 rpm. The profile drill with a diameter of 4.1 mm was set to 300 rpm, while the BLT Tap with a diameter of 4.1 mm was set to a lower speed of 15 rpm.

**Fig. 1:**
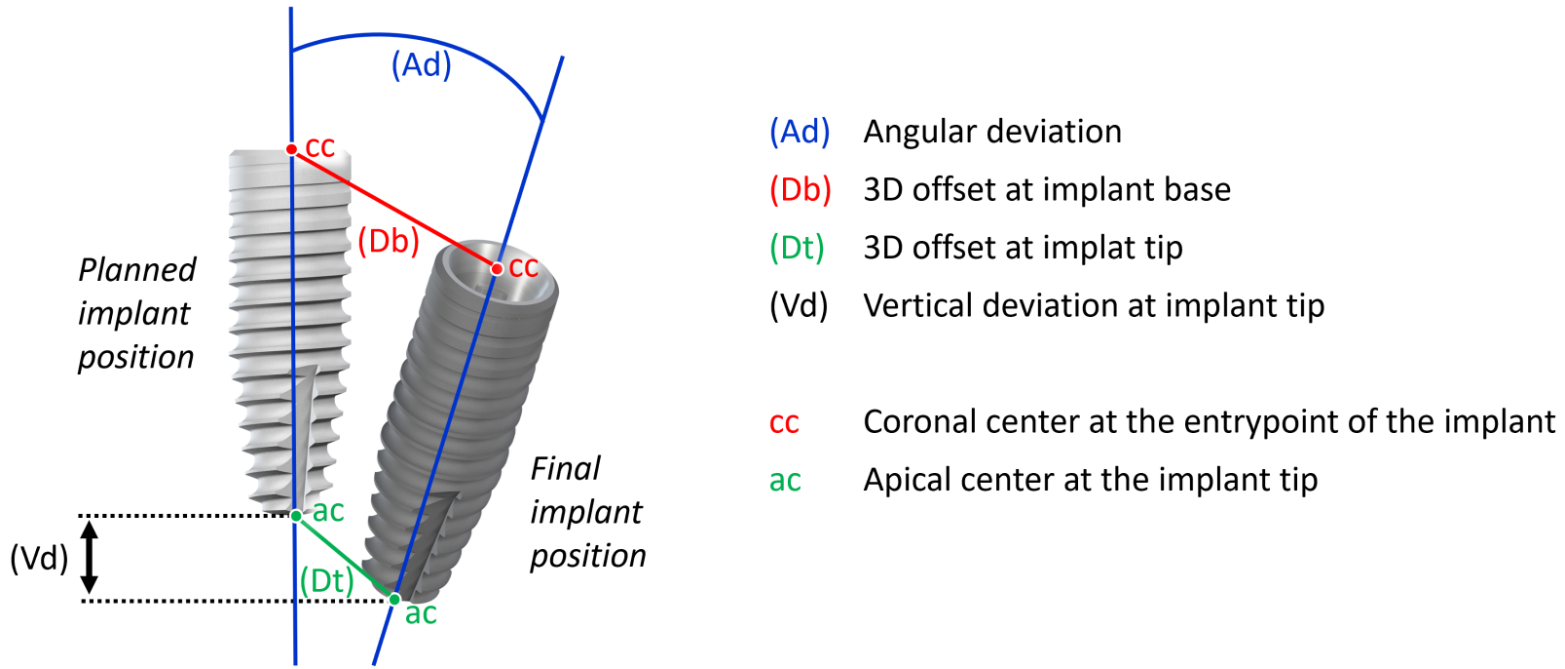
– Evaluated deviation measurements

For the accuracy analysis, RC Mono Scanbodies (Straumann Group, Basel, CH) were mounted on the implants and scanned with the previously used desktop scanner. The 36 surface scans were exported as STL files and compared with the reference data set of the originally created planning case with the planning software. For this purpose the manufacturer of the planning software provides an non-commercially available plug-in tool named Treatment Evaluation. In accordance to the methodology of previously conducted studies [5, 12, 13] four clinically relevant deviation parameters measured between the planned and the final implant position were determined: the three-dimensional offset of the implant base (Db) and the implant tip (Dt) was defined as the distance between the coronal / apical centers of the planned and finally placed implants. The angular deviation (Ad) was calculated as the angle between the longitudinal axes of the planned and the finally placed implant. The vertical deviation at the implant tip (Vd) was calculated as the distance between the apical center of the planned implant and the apical center of the finally placed implant (Fig. 1).

The complete data acquisition, scanning of the models and the implant position evaluation were performed by the investigator using a blinded design.

The values for the angular deviation were calculated by the evaluation software in °(degrees) with one decimal place, the 3D offset and vertical deviation in millimeters with two decimal places. Data were visualized using group-specific boxplots and dotplots. The boxplots visualize five summary measures: the median, the 25th and 75th percentiles (Q1 and Q3), and the upper and lower adjacent values, which are the most extreme values within Q3 + 1.5(Q3-Q1) and Q1-1.5*(Q3-Q1), respectively. The dots represent single values and the red lines mark mean values. Pairwise comparisons between groups were conducted using the Mann-Whitney U test. Furthermore, to assess for each group whether the difference between regions was equal to 0, Wilcoxon signed rank tests were performed. Due to the exploratory character of the study, P-values are to be interpreted only descriptively, thus no formal adjustment for multiple testing was performed. P-values smaller than 0.05 were considered to be statistically significant. Statistical analyses were conducted using Stata/SE 16.0 (StataCorp LLC, Texas, USA).

## Results

### Deviation between groups

All 36 operators were able to complete the experimental implantations with two implants each and all 72 implant positions were submitted to the statistical analysis. For the group comparison regarding the mean TA, the values of the inter-dental gap situation at region 15 and the free-end situation at region 26 were combined and the operator groups were then compared with regard to the respective deviation parameters.

**Fig. 2:**
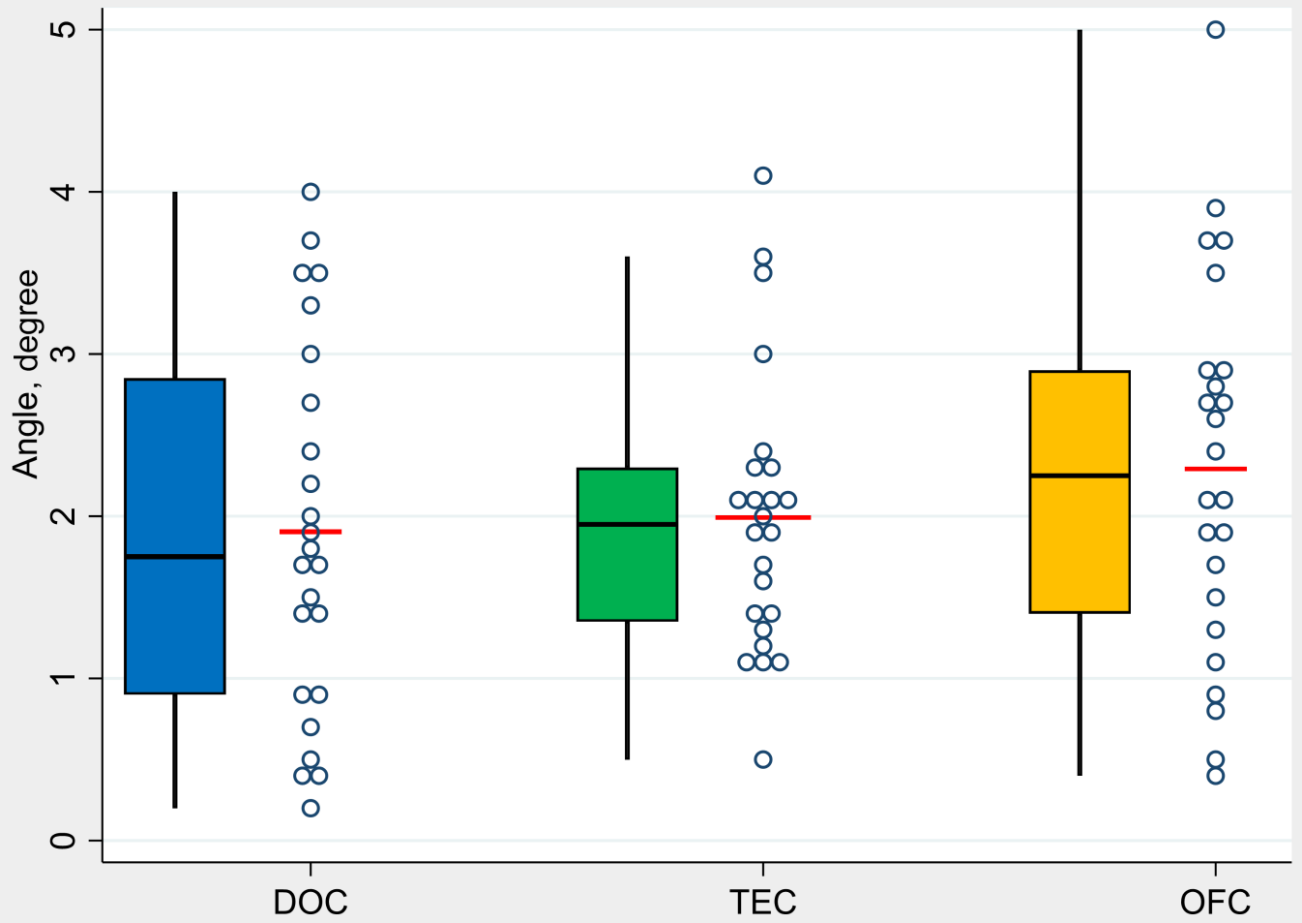
- Boxplots demonstrate analysis of the values in terms of axial deviation (Ad) by groups (*N* = 72 implants)

The mean value of angular deviation (Ad) at control group DOC was 1.9° (min. 0.2° / max. 4.0°) at test group TEC 2.0° (min. 0.5° / max. 4.1°), and for the test group OFC at 2.3° (min. 0.4° / max. 5.0°) (Fig. 2).

In the group comparison, no statistically significant differences were found between the control group DOC and the test groups TEC (*p* = 0.642) and OFC (*p* = 0.348). The comparison between the two test groups also showed no statistically significant difference (*p* = 0.257).

**Fig. 3:**
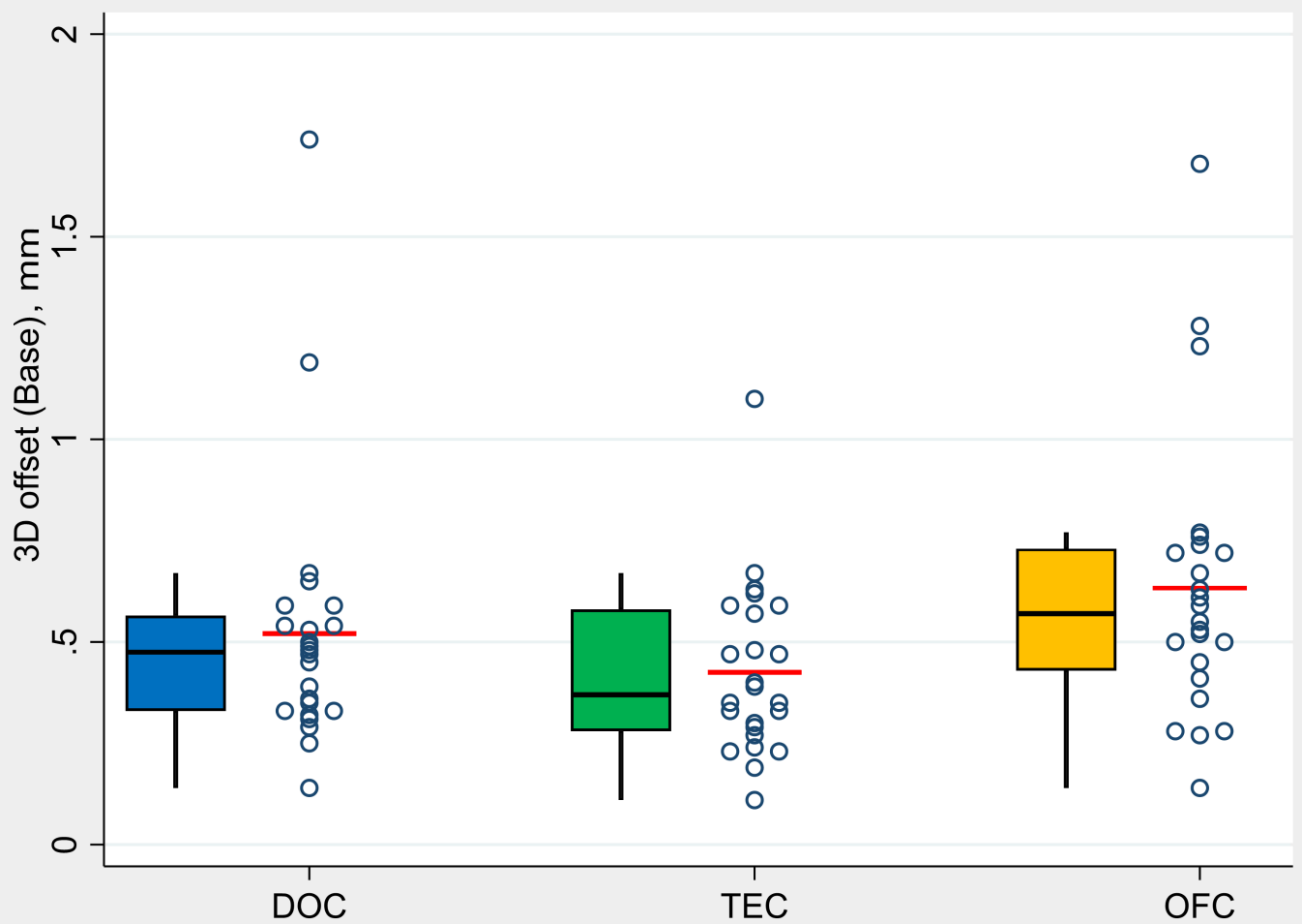
- Boxplots demonstrate analysis of the values in terms of 3-dimensional deviation at implant base (Db) by groups (*N* = 72 implants)

The mean values of the 3-dimensional deviation at implant base (Db) at control group DOC was 0.52 mm (min. 0.14 mm / max. 1.74 mm), at test group TEC 0.42 mm (min. 0.11 mm / max. 1.10 mm), at test group OFC 0.63 mm (min. 0.14 mm / max. 1.68 mm) (Fig. 3).

**Fig. 4:**
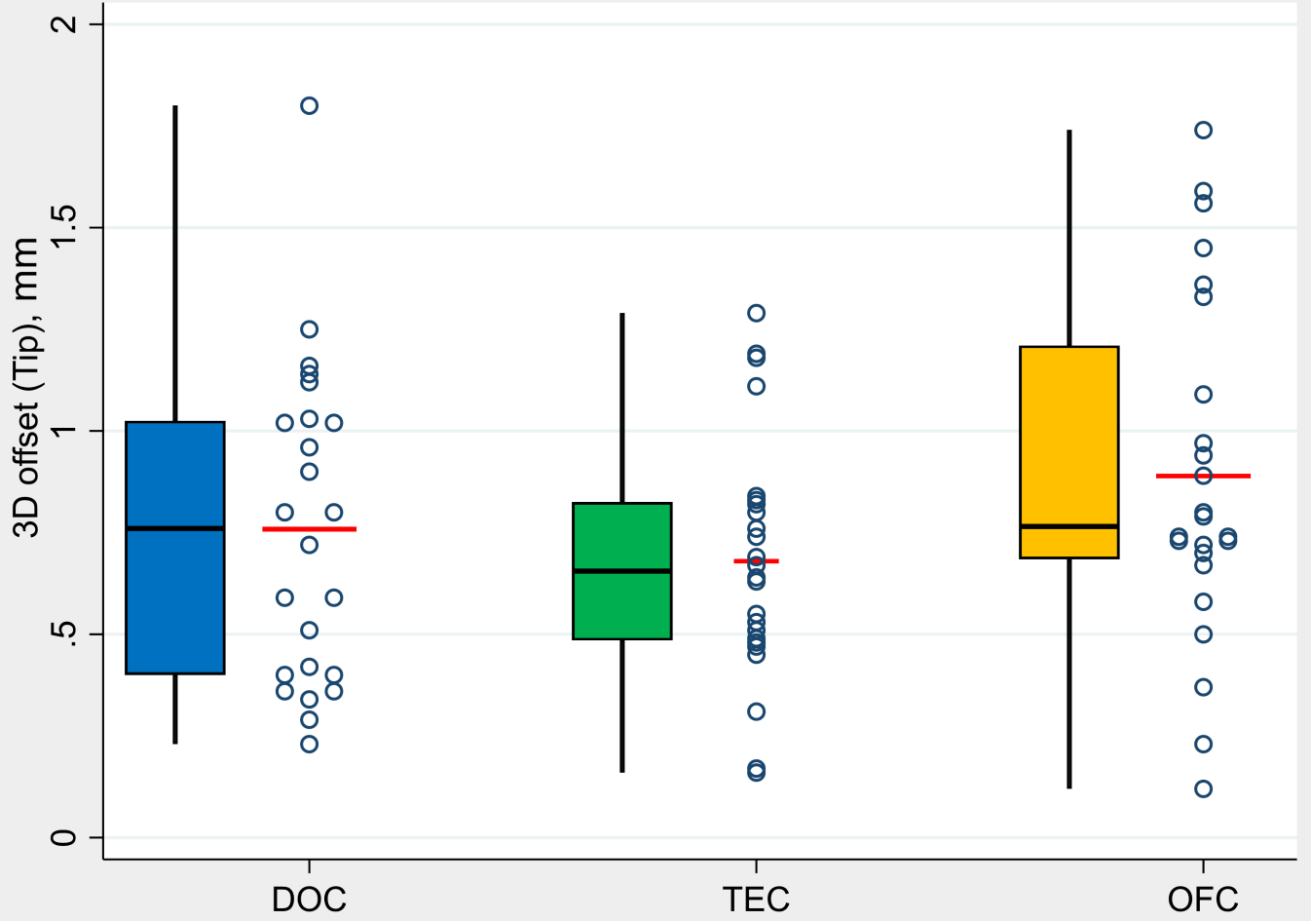
- Boxplots demonstrate analysis of the values in terms of 3-dimensional deviation at implant tip (Dt) by groups (*N* = 72 implants)

The measured mean value of the 3-dimensional deviation at the implant tip (Dt) at control group DOC was 0.76 mm (min. 0.23 mm / max. 1.80 mm), at test group TEC 0.68 mm (min. 0.16 mm / max. 1.29 mm) and at test group OFC 0.89 mm (min. 0.12 mm / max. 1.74) (Fig. 4).

Considering the group comparison, no statistically significant differences were found between the control group DOC and the test groups TEC (*p* = 0.709) and OFC (*p* = 0.381). The comparison between the two test groups also showed no statistically significant difference in terms of this TA parameter (*p* = 0.070).

**Fig. 5:**
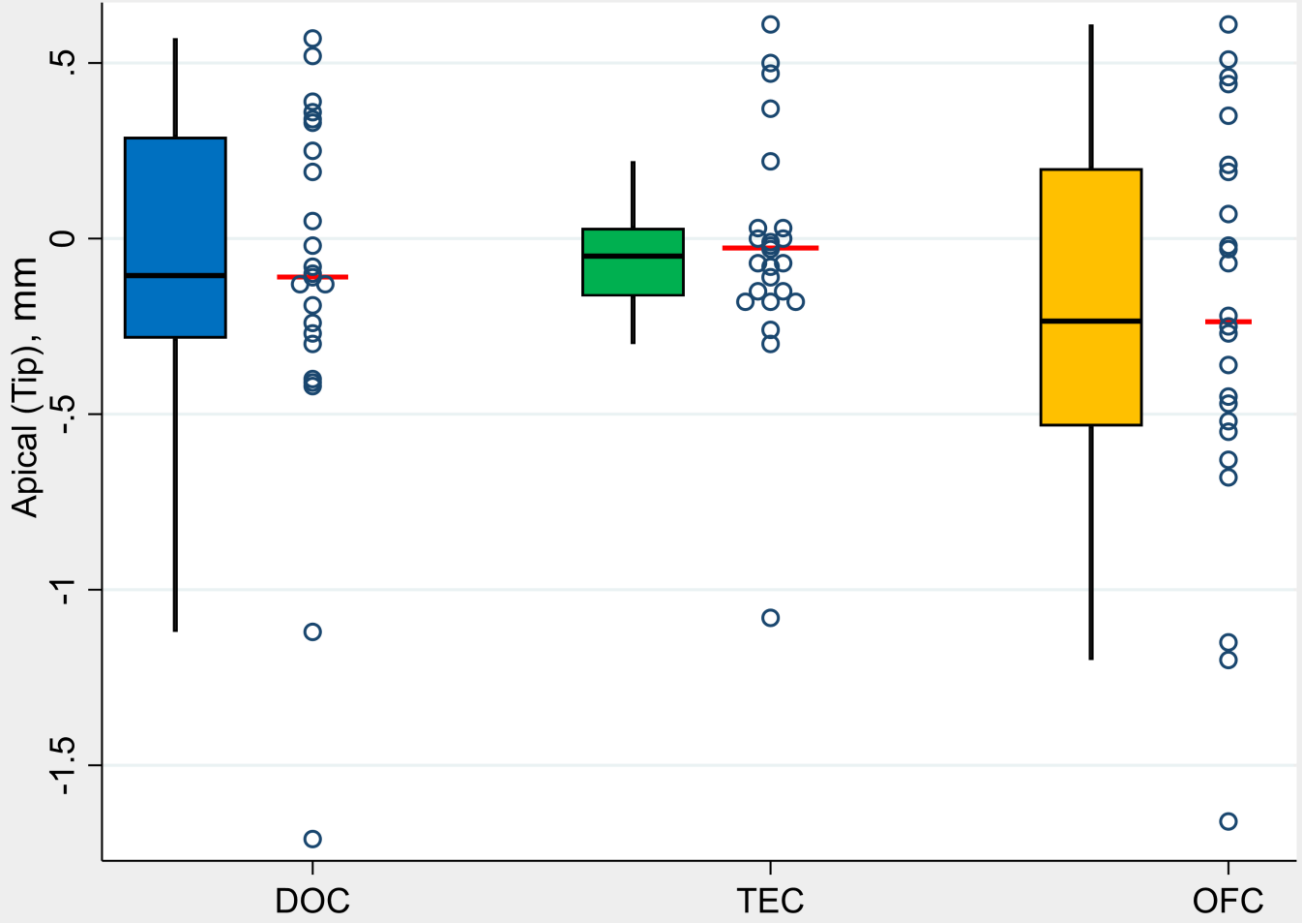
- Boxplots demonstrate analysis of the values in terms of apical (vertical) deviation (Vd) by groups (*N* = 72 implants)

The measured mean value of the vertical deviation at the implant tip (Vd) at the control group DOC was − 0.11 mm (-1.71 mm / max. 0.57 mm), at test group TEC − 0.03 mm (min. -1.08 / max. 0.61 mm), at test group OFC − 0.24 mm (min. -1.66 mm / max. 0.61 mm) (Fig. 5).

Negative values mean a deviation in the coronal direction and positive values mean a deviation in the apical direction. When comparing groups, no statistically significant differences were found between the control group DOC and the test groups TEC (*p* = 0.490) and OFC (*p* = 0.365). The comparison between the two test groups also showed no statistically significant difference in TA (*p* = 0.149) (Table 2).

**Table 2:**
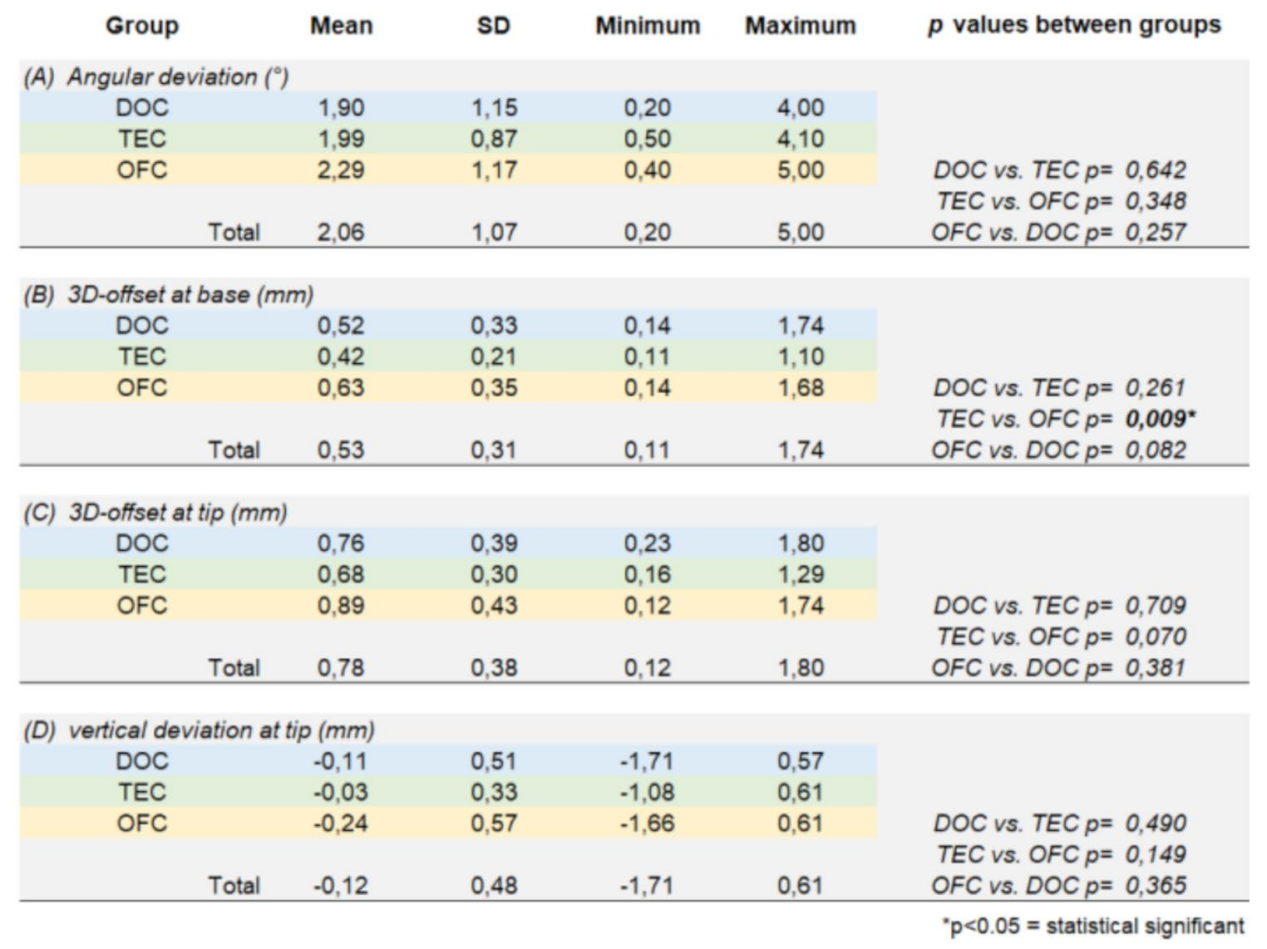
Deviation values in terms of accuracy and comparison between groups

### Deviation between inter-dental gap situation and free end situation

Different values of TA were found between the inter-dental gap and the free end situation. When comparing the mean values of TA regarding the angular deviation it was noticeable that for all three groups of operators the deviations in the free-end situation were larger than in the inter-dental gap situation. These differences turned out to be statistically significant in all three groups (DOC *p* = 0.046, TEC *p* = 0.029, OFC *p* = 0.006).

Comparing the 3D offset at implant base, the groups DOC and OFC achieved smaller deviations in the free-end situation than in the inter-dental gap, whereas TEC achieved larger deviations. However, no statistically significant difference was found for any group (*p* > 0.05).

Comparing the 3D offset at implant tip, it became evident that all three groups of operators achieved greater deviations in the mean values in the free-end situation than in the inter-dental gap situation. The difference in mean values between regions 15 and 26 was found to be statistically significant at TEC (*p* = 0.012).

Regarding the comparison of the vertical deviation at the implant tip between the inter-dental gap and the free-end situation, a shift of the mean values from the negative range in region 15 to the positive range in region 26 was observed in all three groups of operators. The differences between region 15 and region 26 reached statistical significance within the groups DOC (*p* = 0.008) and OFC (*p* = 0.004) (Table 3).

**Table 3:**
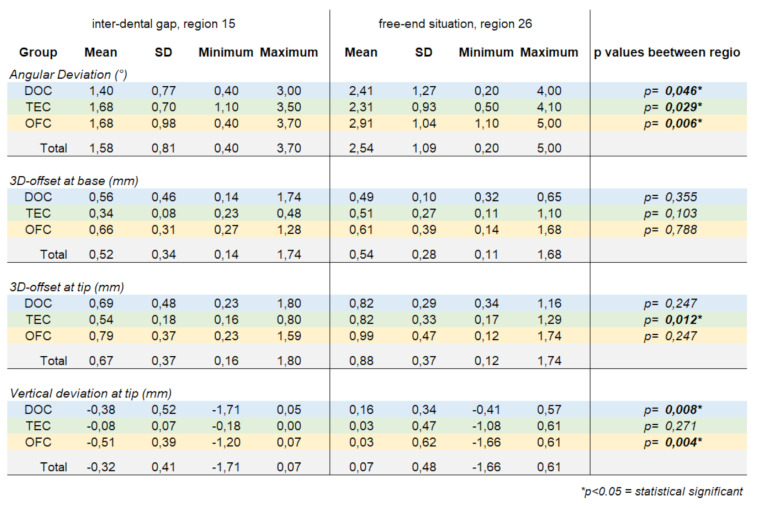
Distribution of deviation at inter-dental gap situation and free end situation

## Discussion

To the authors’ current knowledge, no comparable studies have been conducted on this topic using operators without any relation to dentistry as a test group. In the present study, TA of experimental implantations was compared between experienced clinicians and non-specialist groups. In various current studies, operators related to dentistry are often included, such as dental students or inexperienced surgeons [7, 8, 10, 11, 14]. Product-related user tests with inexperienced or even non-specialist users are often part of a standardized development and validation process, concerning the usability of a product or system. During the initial planning phase of the study, comparing the results between male and female operators was considered since previously published studies have suggested that female surgeons achieved better results [15, 16] or may have superior fine motor skills [17], which could be reflected in the results of transfer accuracy. For this reason, all groups were created with an equal number of male and female subjects. In order to only focus on the influence on clinical experience and expertise on transfer accuracy, the participants were chosen according to a balanced gender ratio. The aim of the present study is to analyze the influence of the user’s competence level on TA of guided implant surgery. An investigation with this focus can only be performed as in vitro study design by reproducing a planned patient case and excluding all other influencing factors to the TA e.g. anatomic characteristics of the patient [18]. To avoid bias due to mechanical influences like the use of different drill guide geometries or various guided surgery systems described in further studies [19, 20], the experimental setup also required all operators to perform the implantation with the same drill guide, because the use of different guides could lead to different implant positions [21]. The coDiagnostiX® planning software was used for planning and designing the drill template. This is an established system that has been used in everyday clinical practice for several years and has been investigated in numerous studies [10, 22–25].

In order to compare the final implant position with the originally planned reference positions, different measurement methods are described in the literature. Currently the most common method to determine the TA is to perform postoperative CTs, which are processed in planning software. The method used in this study to measure the TA with scan bodies, is described in recent studies [6, 19, 26], which also avoids unnecessary radiation exposure and provides a more precise measurement to achieve and compare the implant position [13]. There are several factors that can cause errors in transfer accuracy, such as differences in imaging quality of CBCT and surface scans [27, 28], also issues with the superimposing process and errors of reproducibility when overlaying the different imaging data sets [29–31]. Additionally, the reproducibility of results between the planned and actual placed implant position can be influenced by different production techniques and material selection of the 3D-printed surgical guides [7, 21, 32].

The results of the present study revealed that no statistical significance of the TA could be determined for the deviation parameters when comparing the control group DOC with the two test groups TEC and OFC. Only the 3D offset at implant base revealed a statistical significance (*p* = 0.009) between the test groups TEC and OFC. The results of the present in vitro study also showed that experience has no influence with regard to the TA. Comparing with similar in vitro studies it comes to light that the TA measured in this present study is comparable in terms of magnitude and shows within several parameters even higher accuracy [8, 10, 11, 14, 26, 33, 34].

The fact that even the operators without any expertise and experience in dental implant placement achieved acceptable results regarding TA can be explained by the use of technical guidance through demonstrating a training video. In this case the use of such an animated video tutorial, which demonstrates the complex process steps of the static navigation system, seems to be a proven training tool even for people outside the field or inexperienced operators. At this point, further investigation is recommended, especially on the part of the responsible manufacturers and providers of static navigation systems. Providing the instruction as an animated video tutorial could guide the experienced but also inexperienced surgeon before and during intra-operative use and could provide additional safety.

### Observed complications

**Fig. 6:**
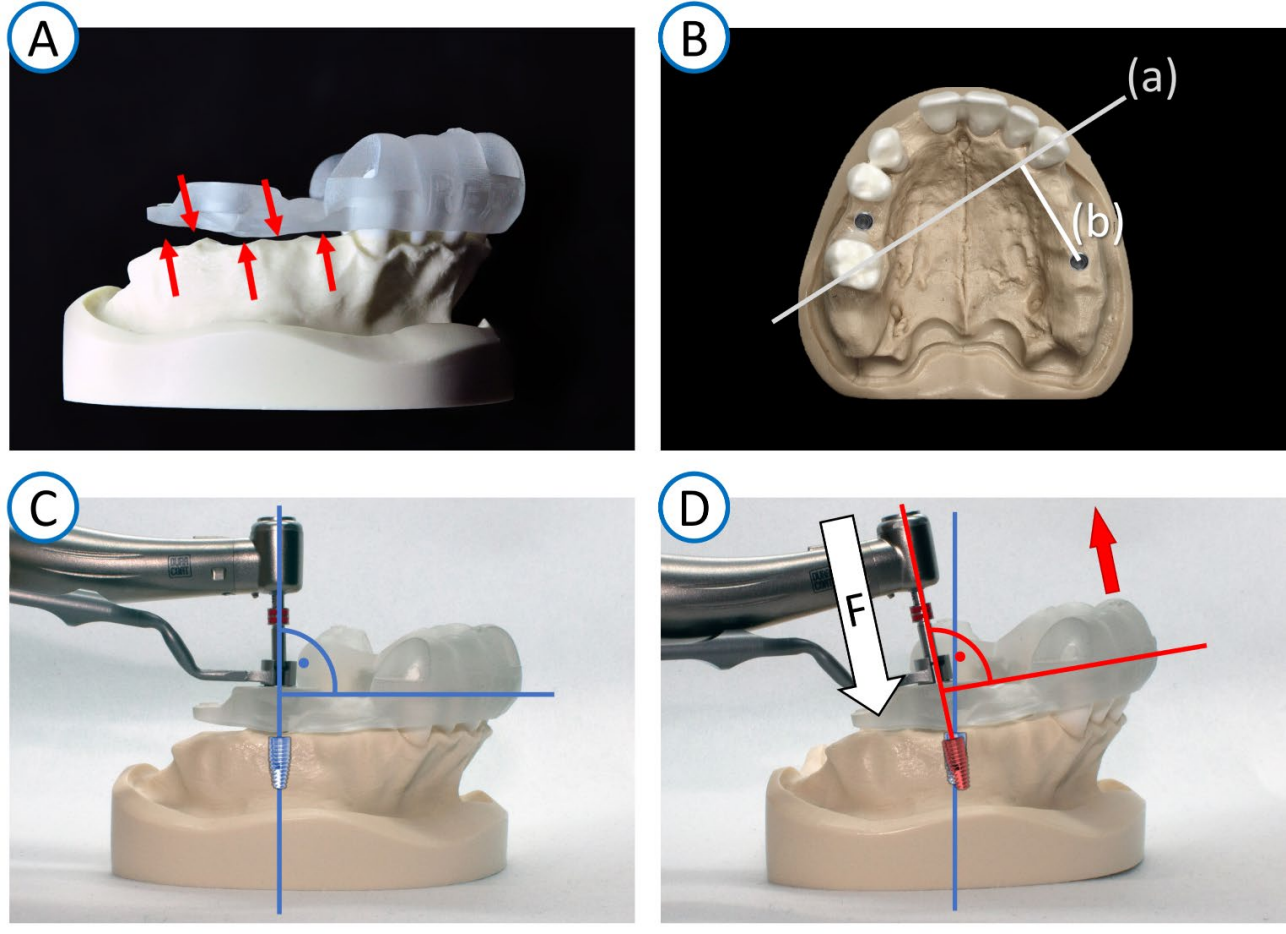
- **A**: The template does not rest basally in the distal area of the free-end situation. **B**: Representation of the rotation axis (a) and lever with force (b). **C**: The template in correct position leads to a correct implant position (blue implant). **D**: Force ‘F’ exerted on the template results in tilting effect. As a result, the template is no longer seated in the correct position, which leads to a deviated implant position (red implant)

Axial deviations can be caused by different reasons. Firstly, it was observed that the surgical template could tilt and rotate during use, which can lead to a deviating angle and thus negatively influence the TA. This phenomenon was observed in all of the three groups during the operator trials performed. On the other hand, the phenomenon of fit tolerances between the sleeves and drill handles discussed by Cassetta et al. (2015) can significantly influence the TA [35]. Significantly larger angular deviations of the implant axis were found for the inserted implants in the free-end situation compared to the inter-dental gap situation. The difference was statistically significant in all three groups of operators (p˂0.05). This significantly different TA can be explained by the intrinsic mobility and especially caused by the extension of the drill template. Whereas the drill template could be supported mesially and distally by teeth in the inter-dental gap situation in region 15, the drill template tended to descend distally in the unilateral free-end situation in region 26. This confirms the results of Toyoshima et al. (2015) and Naziri et al. (2016) that extensive unsupported drill templates in free-end situations may tilt apically and thus have an influence on the TA [36, 37] (Fig. 6).

The mechanism of tilting can be counteracted by actively holding the template in the correct position during use, e.g. by applying finger pressure to the tooth-supported area. However, this method requires a certain level of expertise and practical experience on the part of the user. Due to the experience level it was assumed that the group DOC would achieve a higher TA on these parameters than the test groups. However, this assumption could not be confirmed by the results.

## Conclusion

The present in vitro results showed no statistically significant differences between the control group DOC and the test groups TEC and OFC regarding transfer accuracy at all four deviation parameters investigated (p˃0.05).

In the present in vitro study, clinical expertise and practical experience had no significant effect on transfer accuracy.

However, it is important to emphasize that the present results were obtained in a highly controlled in vitro experimental set up investigating only single procedures of a specific guided surgery workflow and therefore the clinical relevance needs to be critically discussed.

## Data Availability

The datasets used and/or analysed during the current study are available from the corresponding author on reasonable request.

## References

[1] Bover-Ramos F, Viña-Almunia J, Cervera-Ballester J, Peñarrocha-Diago M, García-Mira B (2018) Accuracy of Implant Placement with Computer-guided surgery: a systematic review and Meta-analysis comparing cadaver, clinical, and in Vitro studies. Int J Oral Maxillofac Implants 33(1):101–115 28632253 10.11607/jomi.5556

[2] Gargallo-Albiol J, Barootchi S, Marqués-Guasch J, Wang HL (2020) Fully guided Versus Half-guided and Freehand Implant Placement: systematic review and Meta-analysis. Int J Oral Maxillofac Implants 35(6):1159–1169 33270056 10.11607/jomi.7942

[3] Gargallo-Albiol J, Barootchi S, Salomó-Coll O, Wang HL (2019) Advantages and disadvantages of implant navigation surgery. A systematic review. Annals Anat = Anatomischer Anzeiger: Official Organ Anatomische Gesellschaft 225:1–10 10.1016/j.aanat.2019.04.005 31063802

[4] Schnutenhaus S, Brunken L, Edelmann C, Dreyhaupt J, Rudolph H, Luthardt RG (2020) Alveolar ridge preservation and primary stability as influencing factors on the transfer accuracy of static guided implant placement: a prospective clinical trial. BMC Oral Health 20(1):178 32600405 10.1186/s12903-020-01155-x PMC7322921

[5] Schelbert T, Gander T, Blumer M, Jung R, Rücker M, Rostetter C (2019) Accuracy of computer-guided template-based Implant surgery: a computed tomography-based clinical Follow-Up study. Implant Dent 28(6):556–563 31517650 10.1097/ID.0000000000000936

[6] Abduo J, Lau D (2020) Accuracy of static computer-assisted implant placement in anterior and posterior sites by clinicians new to implant dentistry: in vitro comparison of fully guided, pilot-guided, and freehand protocols. Int J Implant Dentistry 6(1):10 10.1186/s40729-020-0205-3 PMC7064711 32157478

[7] Schulz MC, Hofmann F, Range U, Lauer G, Haim D (2019) Pilot-drill guided vs. full-guided implant insertion in artificial mandibles-a prospective laboratory study in fifth-year dental students. Int J Implant Dentistry 5(1):23 10.1186/s40729-019-0176-4 PMC6593025 31240421

[8] Van de Wiele G, Teughels W, Vercruyssen M, Coucke W, Temmerman A, Quirynen M (2015) The accuracy of guided surgery via mucosa-supported stereolithographic surgical templates in the hands of surgeons with little experience. Clin Oral Implants Res 26(12):1489–1494 25318961 10.1111/clr.12494

[9] Park SJ, Leesungbok R, Cui T, Lee SW, Ahn SJ (2017) Reliability of a CAD/CAM Surgical Guide for Implant Placement: an in Vitro comparison of surgeons’ experience levels and Implant sites. Int J Prosthodont 30(4):367–169 28697207 10.11607/ijp.5179

[10] Kivovics M, Pénzes D, Németh O, Mijiritsky E (2020) The Influence of Surgical Experience and Bone Density on the Accuracy of Static Computer-Assisted Implant Surgery in Edentulous Jaws Using a Mucosa-Supported Surgical Template with a Half-Guided Implant Placement Protocol-A Randomized Clinical Study. Materials (Basel, Switzerland). ;13(24) 10.3390/ma13245759 PMC7765911 33348589

[11] Cushen SE, Turkyilmaz I (2013) Impact of operator experience on the accuracy of implant placement with stereolithographic surgical templates: an in vitro study. J Prosthet Dent 109(4):248–254 23566606 10.1016/S0022-3913(13)60053-0

[12] Bilhan H, Arat S, Mumcu E, Geckili O, Sakar O (2012) Precision of implant placement with stereolithographic templates: a pilot in vitro study. J Oral Implantol 38(5):569–574 21126171 10.1563/AAID-JOI-D-10-00109

[13] Brandt J, Brenner M, Lauer HC, Brandt S (2018) Accuracy of a template-guided Implant surgery system with a CAD/CAM-Based measurement method: an in Vitro Study. Int J Oral Maxillofac Implants 33(2):328–334 29534120 10.11607/jomi.5799

[14] Cassetta M, Bellardini M (2017) How much does experience in guided implant surgery play a role in accuracy? A randomized controlled pilot study. Int J Oral Maxillofac Surg 46(7):922–930 28366450 10.1016/j.ijom.2017.03.010

[15] Tsugawa Y, Jena AB, Figueroa JF, Orav EJ, Blumenthal DM, Jha AK (2017) Comparison of Hospital Mortality and Readmission Rates for Medicare Patients Treated by male vs Female Physicians. JAMA Intern Med 177(2):206–213 27992617 10.1001/jamainternmed.2016.7875 PMC5558155

[16] Wallis CJ, Ravi B, Coburn N, Nam RK, Detsky AS, Satkunasivam R (2017) Comparison of postoperative outcomes among patients treated by male and female surgeons: a population based matched cohort study. BMJ (Clinical Res ed) 359:j4366 10.1136/bmj.j4366 PMC6284261 29018008

[17] Peters M, Servos P, Day R (1990) Marked sex differences on a fine motor skill task disappear when finger size is used as covariate. J Appl Psychol 75(1):87–90 2307635 10.1037/0021-9010.75.1.87

[18] Putra RH, Yoda N, Iikubo M, Kataoka Y, Yamauchi K, Koyama S et al (2020) Influence of bone condition on implant placement accuracy with computer-guided surgery. Int J Implant Dentistry 6(1):62 10.1186/s40729-020-00249-z PMC7502099 32951152

[19] El Kholy K, Janner SFM, Schimmel M, Buser D (2019) The influence of guided sleeve height, drilling distance, and drilling key length on the accuracy of static computer-assisted Implant surgery. Clin Implant Dent Relat Res 21(1):101–107 30589502 10.1111/cid.12705

[20] Laederach V, Mukaddam K, Payer M, Filippi A, Kühl S (2017) Deviations of different systems for guided implant surgery. Clin Oral Implants Res 28(9):1147–1151 27460679 10.1111/clr.12930

[21] Dalal N, Ammoun R, Abdulmajeed AA, Deeb GR, Bencharit S (2019) Intaglio Surface Dimension and Guide Tube deviations of Implant Surgical guides Influenced by Printing Layer Thickness and Angulation setting. J Prosthodontics: Official J Am Coll Prosthodontists 10.1111/jopr.13138 31886914

[22] Kühl S, Payer M, Zitzmann NU, Lambrecht JT, Filippi A (2015) Technical accuracy of printed surgical templates for guided implant surgery with the coDiagnostiX ™ software. Clin Implant Dent Relat Res 17(Suppl 1):e177–e182 24020645 10.1111/cid.12152

[23] Derksen W, Wismeijer D, Flügge T, Hassan B, Tahmaseb A (2019) The accuracy of computer-guided implant surgery with tooth-supported, digitally designed drill guides based on CBCT and intraoral scanning. A prospective cohort study. Clin Oral Implants Res 30(10):1005–1015 31330566 10.1111/clr.13514

[24] Flügge T, Ludwig U, Hövener JB, Kohal R, Wismeijer D, Nelson K (2020) Virtual implant planning and fully guided implant surgery using magnetic resonance imaging-Proof of principle. Clinical oral implants research 10.1111/clr.13592 32105363

[25] Kiatkroekkrai P, Takolpuckdee C, Subbalekha K, Mattheos N, Pimkhaokham A (2020) Accuracy of implant position when placed using static computer-assisted implant surgical guides manufactured with two different optical scanning techniques: a randomized clinical trial. Int J Oral Maxillofac Surg 49(3):377–383 31543382 10.1016/j.ijom.2019.08.019

[26] Lin C-C, Ishikawa M, Huang B-H, Huang M-S, Cheng H-C, Maida T et al (2020) In Vitro Accuracy of Static guided Implant surgery measured by Optical scan: examining the impact of Operator Experience. Appl Sci 10(8):2718

[27] Dreiseidler T, Tandon D, Kreppel M, Neugebauer J, Mischkowski RA, Zinser MJ et al (2012) CBCT device dependency on the transfer accuracy from computer-aided implantology procedures. Clin Oral Implants Res 23(9):1089–1097 22680780 10.1111/j.1600-0501.2011.02272.x

[28] Ender A, Zimmermann M, Mehl A (2019) Accuracy of complete- and partial-arch impressions of actual intraoral scanning systems in vitro. Int J Comput Dent 22(1):11–19 30848250

[29] Horwitz J, Zuabi O, Machtei EE (2009) Accuracy of a computerized tomography-guided template-assisted implant placement system: an in vitro study. Clin Oral Implants Res 20(10):1156–1162 19519787 10.1111/j.1600-0501.2009.01748.x

[30] Choi YD, Mai HN, Mai HY, Ha JH, Li LJ, Lee DH (2020) The effects of distribution of image matched Fiducial markers on accuracy of computer-guided Implant surgery. J Prosthodontics: Official J Am Coll Prosthodontists 29(5):409–414 10.1111/jopr.13171 32237001

[31] Nickenig HJ, Eitner S, Rothamel D, Wichmann M, Zöller JE (2012) Possibilities and limitations of implant placement by virtual planning data and surgical guide templates. Int J Comput Dent 15(1):9–21 22930944

[32] Henprasert P, Dawson DV, El-Kerdani T, Song X, Couso-Queiruga E, Holloway JA (2020) Comparison of the Accuracy of Implant position using Surgical guides fabricated by additive and subtractive techniques. J Prosthodontics: Official J Am Coll Prosthodontists 10.1111/jopr.13161 32147893

[33] Rungcharassaeng K, Caruso JM, Kan JY, Schutyser F, Boumans T (2015) Accuracy of computer-guided surgery: a comparison of operator experience. J Prosthet Dent 114(3):407–413 26119019 10.1016/j.prosdent.2015.04.004 PMC4486338

[34] Fernández-Gil Á, Gil HS, Velasco MG, Moreno Vázquez JC (2017) An in Vitro Model to evaluate the accuracy of guided Implant Placement based on the Surgeon’s experience. Int J Oral Maxillofac Implants 32(3):151–154 28494035 10.11607/jomi.5024

[35] Cassetta M, Di Mambro A, Di Giorgio G, Stefanelli LV, Barbato E (2015) The influence of the tolerance between Mechanical Components on the Accuracy of implants inserted with a Stereolithographic Surgical Guide: a retrospective clinical study. Clin Implant Dent Relat Res 17(3):580–588 23879723 10.1111/cid.12120

[36] Toyoshima T, Tanaka H, Sasaki M, Ichimaru E, Naito Y, Matsushita Y et al (2015) Accuracy of implant surgery with surgical guide by inexperienced clinicians: an in vitro study. Clin Experimental Dent Res 1(1):10–17 10.1002/cre2.3 PMC5839200 29744135

[37] Naziri E, Schramm A, Wilde F (2016) Accuracy of computer-assisted implant placement with insertion templates. GMS Interdisciplinary Plast Reconstr Surg DGPW 5:Doc15 10.3205/iprs000094 PMC4889312 27274440

