# Correction to: Influence of clinical expertise and practical experience on transfer accuracy in guided dental implant placement - an in vitro study

**DOI:** 10.1007/s10006-025-01480-x

**Published:** 2025-10-23

**Authors:** Florian Sebastian Reiff, Charlotte Bischoff, Henriette Woelfler, Stefan Roehling

**Affiliations:** 1 Center of Oral Health, University of Greifswald, Greifswald, Germany; 2 Straumann Group, Freiburg im Breisgau, Germany; 3 Straumann Group, Basel, Switzerland; 4 University of Bamberg, Bamberg, Germany; 5 PD Dr. med. dent./Private Dental Clinic PD Dr. Gahlert and PD Dr. Roehling, Munich, Germany; 6 Clinic for Oral and Cranio-Maxillofacial Surgery, Hightech Research Center, University Hospital Basel, Basel, Switzerland

**Correction to: Oral and Maxillofacial Surgery (2024) 28:1491–1500**

10.1007/s10006-024-01269-4

In the final version of the manuscript, some columns in Tables 2 and 3 are missing from the published article. The source manuscript includes all the columns, but an error occurred during the production process, resulting in the last columns being omitted.

The corrected Tables 2 and 3 are shown below.

Incorrect Table 2


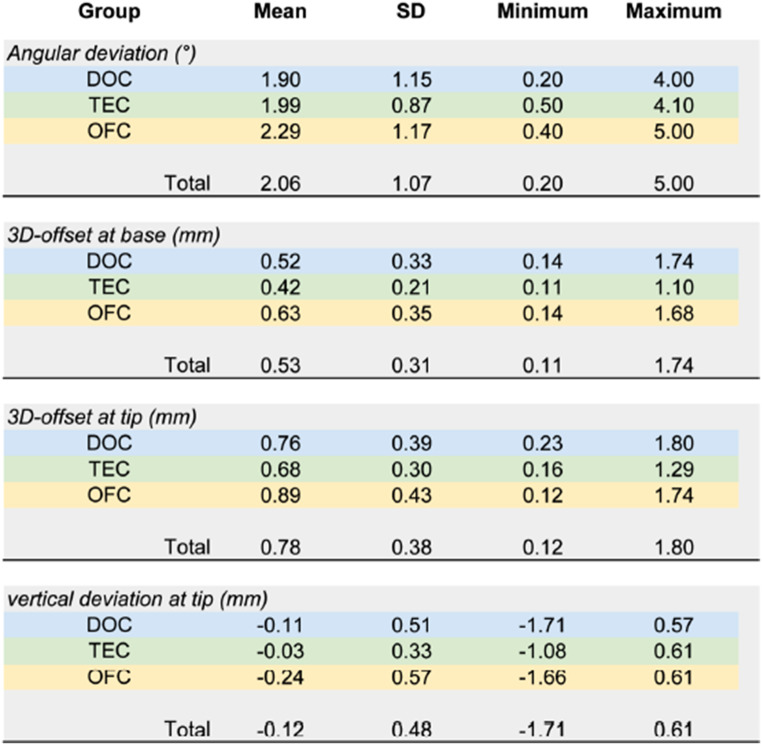


Correct Table 2


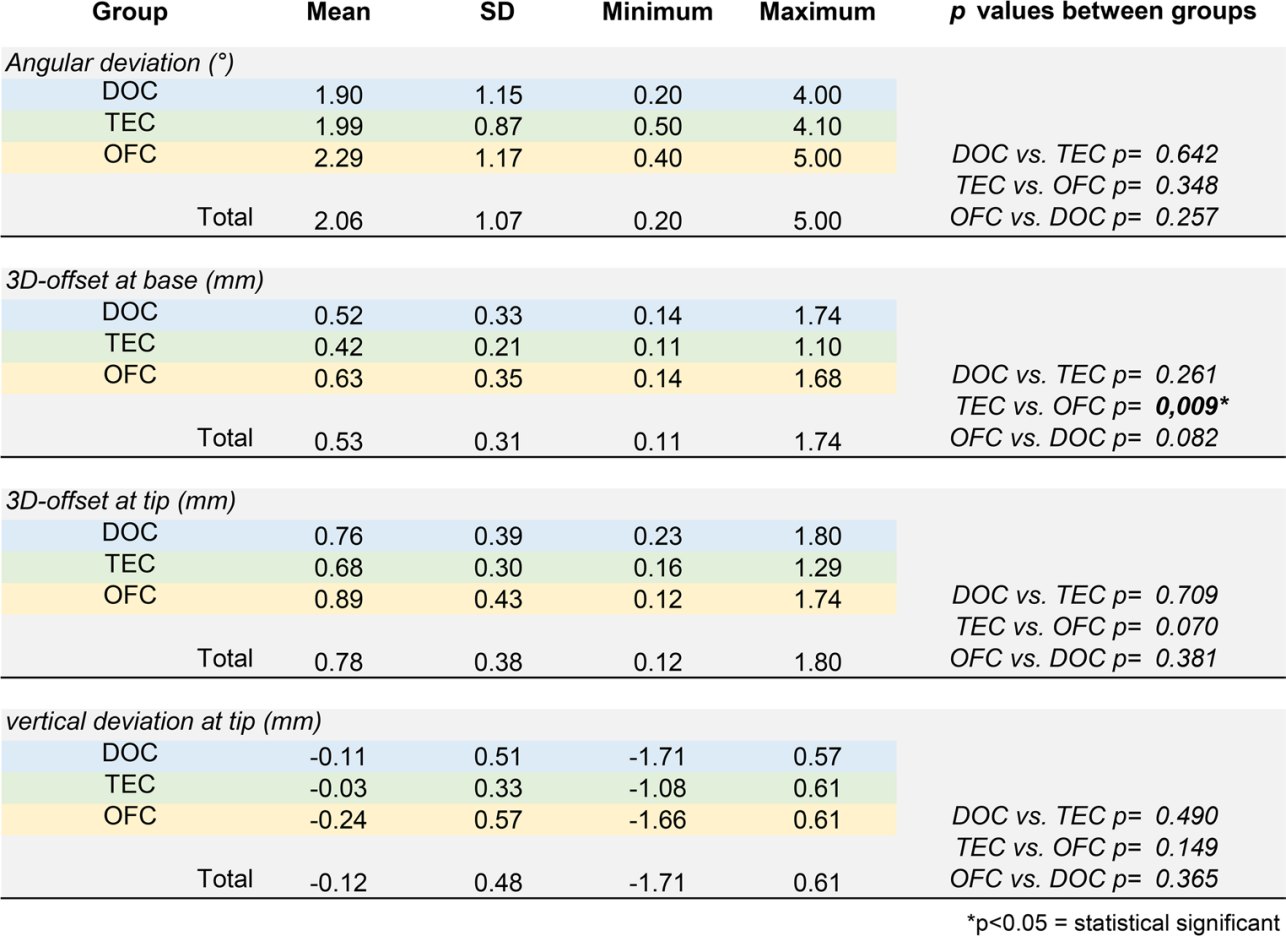


Incorrect Table 3
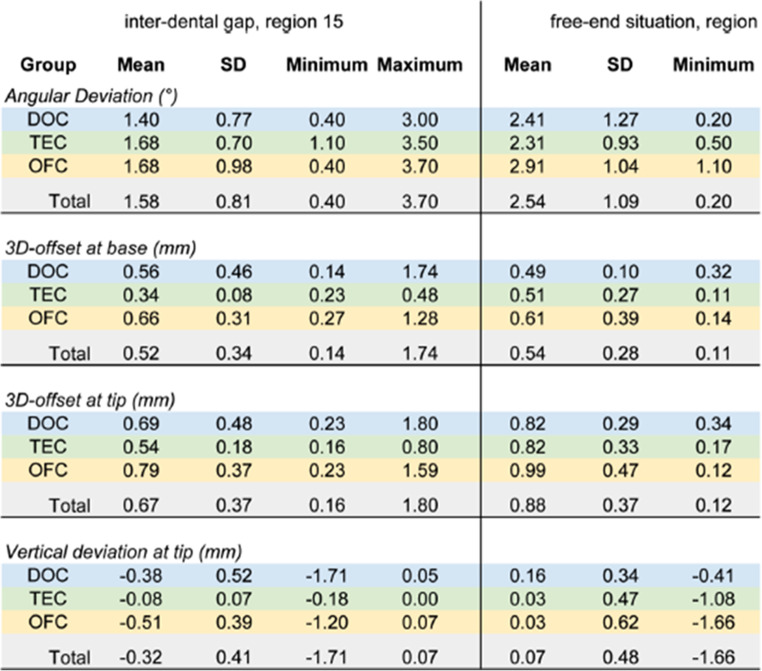


Correct Table 3


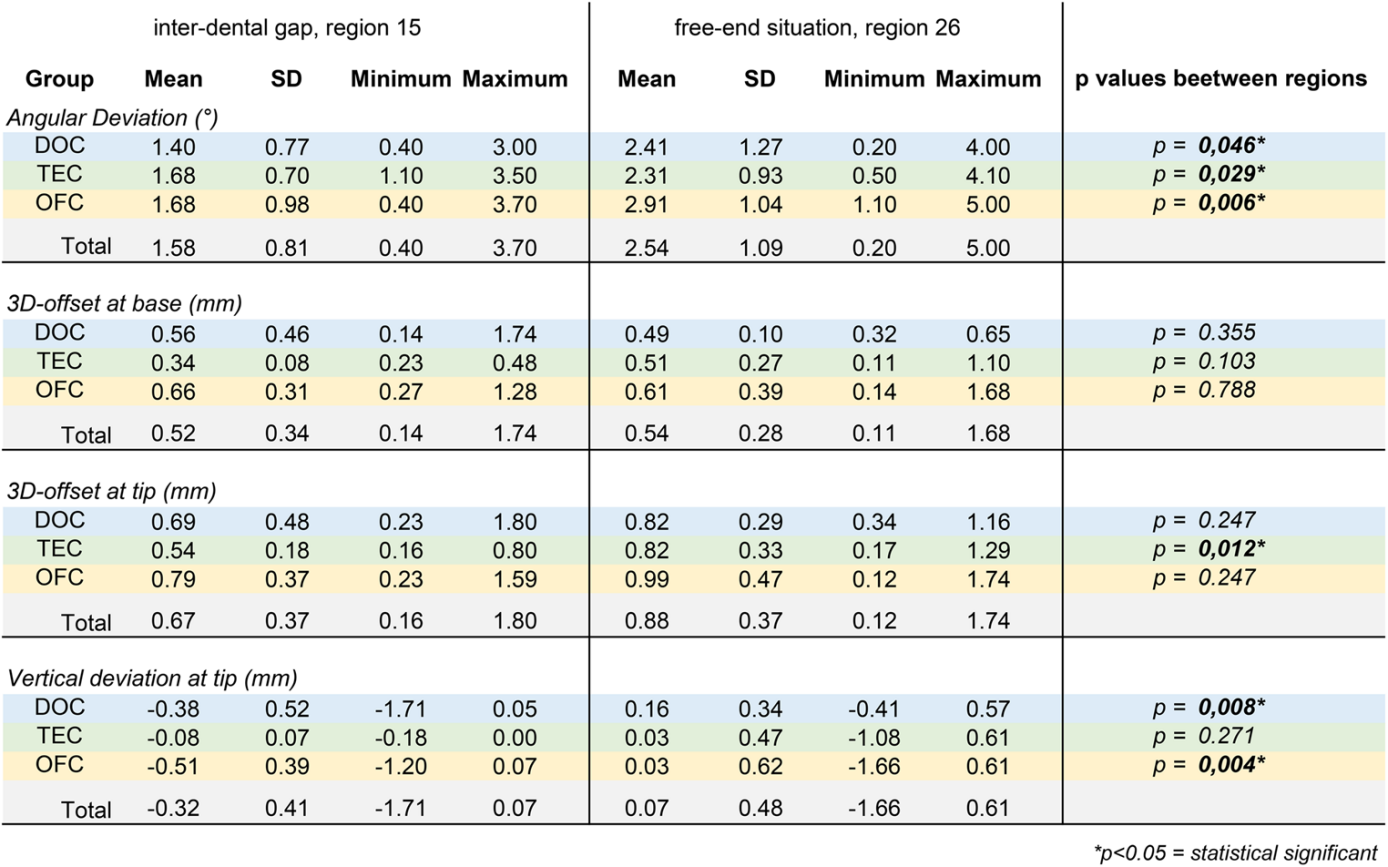


The original article has been corrected.

